# Deciphering signal transduction networks in the liver by mechanistic mathematical modelling

**DOI:** 10.1042/BCJ20210548

**Published:** 2022-06-24

**Authors:** Lorenza A. D’Alessandro, Ursula Klingmüller, Marcel Schilling

**Affiliations:** Division Systems Biology of Signal Transduction, German Cancer Research Center (DKFZ), Im Neuenheimer Feld 280, 69120 Heidelberg, Germany

**Keywords:** cytokines, growth factors, hepatocellular carcinoma, hepatocytes, liver, mathematical modelling

## Abstract

In health and disease, liver cells are continuously exposed to cytokines and growth factors. While individual signal transduction pathways induced by these factors were studied in great detail, the cellular responses induced by repeated or combined stimulations are complex and less understood. Growth factor receptors on the cell surface of hepatocytes were shown to be regulated by receptor interactions, receptor trafficking and feedback regulation. Here, we exemplify how mechanistic mathematical modelling based on quantitative data can be employed to disentangle these interactions at the molecular level. Crucial is the analysis at a mechanistic level based on quantitative longitudinal data within a mathematical framework. In such multi-layered information, step-wise mathematical modelling using submodules is of advantage, which is fostered by sharing of standardized experimental data and mathematical models. Integration of signal transduction with metabolic regulation in the liver and mechanistic links to translational approaches promise to provide predictive tools for biology and personalized medicine.

## Introduction

Cell-to-cell communication is known since the mid-1800, when Claude Bernard developed the concept of *le milieu intérieur*, addressing the concept of molecule secretion and action on a distant organ [1,2]. Since then, efforts have been made to discover secreted signal transduction molecules, such as cytokines and growth factors, and their mode of action to transmit the intracellular signal with consequent biological response.

In this review, we discuss signal transduction in human and rodent cells, as they share similar modalities of cell-to-cell and intracellular communication. For many years, studies on signal transduction focused on individual factors inducing single intracellular cascades. However, complexity vastly increased with the discovery that single factors regulate multiple signal transduction pathways and that the interplay of several ligands, cross-talk and feedback mechanisms regulate the biological output. As an additional layer of complexity, recently awareness increased on the importance of understanding mechanisms that determine dynamic behaviour of signal transduction pathways, such as signal duration, activation kinetic, signal specificity and ultimately biological responses [3]. At this level, several layers of regulations can be distinguished: (i) ligand–receptor interaction, (ii) receptor trafficking; (iii) downstream signal transduction cross-talk and feedback and (iv) transcriptional regulation. To functionally understand the behaviour of signal transduction pathways with complex regulation, mechanistic mathematical modelling based on quantitative data was performed in the examples discussed here. In contrast with statistical modelling, such as machine learning based on big data [4], mechanistic models seek to establish a causal relationship between inputs and outputs. Thus, these models can even be calibrated based on small data sets and, rather than inferring correlations, can be used as predictive tools [5].

**Figure 1.**
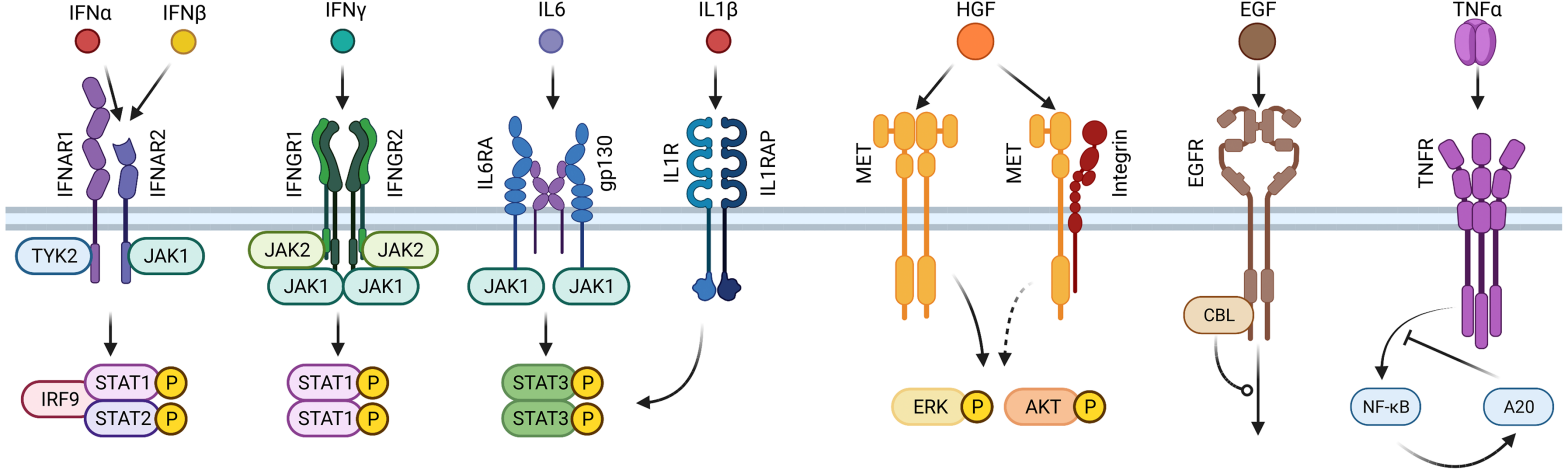
Biological questions addressed with mechanistic mathematical modelling. Cytokines activate identical or overlapping transcription factors; interaction of the HGF receptor MET with other receptors influences its signalling strength; EGF induces internalization of its cognate receptor EGFR; TNFα-induced signal transduction is subject to complex feedback regulation.

Cytokines are small secreted glycoproteins that by binding to specific cell surface receptors initiate a cascade of intracellular signals. Their mode of action can be autocrine, paracrine or endocrine [6]. Their primary functions include the regulation of the immune system, haematopoiesis and developmental processes. In the liver, they play an import part in immunity, inflammation including the acute phase response, liver regeneration, fibrosis and cancer [7]. Here, we focus on interferons (IFN) and interleukins (IL) that are recognized by cytokine receptors. Cytokine receptor chains comprise an extracellular domain that interacts with the cytokine, a single transmembrane domain and a signal-transducing cytoplasmic domain. Upon ligand binding, the cytokine receptor chains on the cell surface associate or are stabilized as dimers or oligomers [8]. The cytoplasmic domain lacks intrinsic kinase activity and associates with a protein tyrosine kinase of the Janus kinase (JAK) family [9]. Tyrosine phosphorylation of the cytoplasmic receptor chains activates several signal transduction components, predominantly proteins of the signal transducer and activator of transcription (STAT) family. Upon recruitment of the STAT proteins via their SH2 domain to the phosphorylated tyrosines of the receptor chains, they in turn are phosphorylated by the JAK kinases. STAT proteins dimerize, enter the nucleus and induce gene transcription. Dephosphorylated STAT proteins translocate back to the cytoplasm and can be phosphorylated again [10]. Among the induced target genes are negative regulators including the suppressor of cytokine signalling (SOCS) proteins [11], which inhibit the kinase activity of the JAK proteins. As examples, we here concentrate on three interferons — the type I interferons IFNα and IFNβ and the type II interferon IFNγ — and two interleukins — IL6 and IL1β (Figure 1).

IFNα can be divided into 13 different subtypes that are encoded by different genes, while there is only one type of IFNβ [12]. In the studies discussed here, mostly IFNα2 encoded by *IFNA2* was used to represent IFNα. Both IFNα and IFNβ preferentially bind to the cytokine receptor subunit interferon alpha/beta receptor (IFNAR)2. Upon binding of the ligand, the other subunit IFNAR1 is recruited and a ternary complex is formed [13]. While IFNAR1 is constitutively associated with the JAK family member TYK2, JAK1 is bound to IFNAR2. Upon activation of these two kinases, STAT1 and STAT2 are recruited to the receptor chains and tyrosine phosphorylated. A unique feature of type I IFN-induced JAK/STAT signal transduction is that STAT1 and STAT2 bind an additional protein, IRF9, to form the ISGF3 complex, which is the main transcription factor complex [14]. In contrast, IFNγ binds to the receptor chains IFNGR1, associated with JAK1, and IFNGR2, associated with JAK2. Ligand binding induces phosphorylation of STAT1, which forms homodimers as active transcription factor complexes [15].

IL6 first binds to the IL6R subunit alpha and this complex then recruits two molecules of the glycoprotein gp130. The major receptor-bound kinase is JAK1 and signal transduction is mediated by STAT3 homodimers [16]. Lastly, IL1β associates with high affinity to its cognate receptor IL1R [17] and upon binding forms a complex with the co-receptor IL1 receptor accessory protein (IL1RAP). Besides activation of the p38 and the NFκB pathways, IL1β was also reported to lead to phosphorylation of STAT3 [18].

Thus, these five cytokines activate identical or overlapping transcription factors in the liver and the mechanisms that ensure that each ligand induces a specific cell fate response are unclear. It is further not yet understood what happens if a hepatocyte encounters the same ligand repeatedly and how the cell reacts if it is stimulated with different ligands simultaneously or sequentially. In the next chapter, we will delineate how experimental data generation in combination with mechanistic mathematical modelling is beginning to answer some of these questions.

Growth factors such as the hepatocyte growth factor (HGF) and the epidermal growth factor (EGF) are recognized by liver cells via receptor tyrosine kinase (RTK) receptors [19]. These transmembrane proteins, in contrast with the cytokine receptors discussed above, harbour intrinsic tyrosine kinase activity. HGF binds to the RTK receptor MET, leading to the phosphorylation of its cytoplasmic domain and activation of various signal transductions pathways including the MAPK and the PI3K/AKT pathway [20]. However, HGF-induced signal transduction is not an isolated cascade. Cross-talk mechanisms that can be initiated at the plasma membrane by the interaction of unrelated receptors as well as at the intracellular level with interaction among different signal transduction cascades vastly increase the complexity of signal transduction. These mechanisms influence the ligand-induced amplitude and duration of the activation of signal transduction [21]. It was shown that MET interacts with other receptors, such as plexins, CD44, FAS, VEGFR, EGFR and integrins, modulating HGF-induced receptor activation and degradation [22]. EGF binds to its cognate receptor EGFR, leading to the phosphorylation of numerous tyrosine residues on its cytoplasmic domain [23], which induce activation of downstream signal transduction components and regulate receptor trafficking, including internalization, recycling and degradation [24]. The final factor discussed here that is involved in functional regulation of the liver is tumour necrosis factor alpha (TNFα). TNFα is decoded by liver cells via the binding of the transmembrane receptor TNF receptor 1 (TNFR1 or p55) or TNF receptor 2 (TNFR2 or p75), with TNFR1 regulating a wider range of functions, including liver regeneration [25]. TNFα acts by activating the intracellular nuclear factor κ-light-chain-enhancer of activated B cells (NFκB) leading to the activation of transcription factor complexes formed by homo or heterodimers of p50, p52, p65, RelB and Rel C subunits [26]. These complexes are retained in the cytoplasm by binding to inhibitors such as IκBα, IκBβ and IκBε. Upon TNFα stimulation, the inhibitory proteins are phosphorylated with subsequent release of the transcription factor complexes, which translocate to the nucleus and activate gene transcription. Among the TNFα target genes there are also regulators of the NFκB pathway, such as IkBα [27] and A20 [28], which are key negative feedback regulators of the pathway. Unresolved questions relate to the molecular mechanisms resulting from the interaction of MET with other receptors and its medical implications, internalization of EGFR depending on the ligand dose and the mechanism of the A20-dependent feedback regulation in the TNFα-induced signal transduction cascade. In the following, we will illustrate how step-wise mechanistic mathematical modelling using submodules was employed to answer these questions.

## Cytokine waves augmenting or diminishing pathway activation in hepatocytes

The liver is targeted by several viruses. Hepatotropic viruses such as hepatitis B virus (HBV) and hepatitis C virus (HCV) are currently still the major risk factors for hepatocellular carcinoma (HCC) [29]. Type I interferons such as interferon alpha (IFNα) and interferon beta (IFNβ) act as the first line of defence of the hepatocytes against hepatotropic viruses [30]. Type I IFNs play a vital role in innate immune responses against viruses and have an immunomodulatory effect on natural killer cells, macrophages and dendritic cells [31]. IFNα and IFNβ are produced in most cell types upon viral or microbial infection. The presence of viruses in cells is perceived by pattern recognition sensors, which detect conserved pathogen-associated molecular patterns, e.g. viral RNA and DNA. Via different signal transduction pathways, including AP-1, IRF3/IRF7 and NFκB, these sensors induce the expression of IFNα and IFNβ [32]. IRF7 is required for an efficient expression of IFNα, but is not essential for the induction of IFNβ [33]. Because IRF7 concentrations are initially low, the first increase in interferon production mainly consists of IFNβ. To disentangle the molecular events upon dynamic changes of interferon production in the liver, the question arises how the antiviral response of hepatocytes is altered if the cells have previously been exposed to interferon. Quantitative immunoblotting experiments performed in hepatoma cells and primary human hepatocytes showed that pre-stimulation with a low dose of IFNα hypersensitizes the pathway, i.e. the antiviral response is stronger than in cells that were not pre-stimulated [34]. However, pre-stimulation with a high dose of IFNα desensitized the pathway, i.e. the cells were no longer responsive to stimulation. Mathematical modelling revealed that hypersensitization was caused by the positive feedback protein IRF9. In contrast, high doses of IFNα induced the negative feedback proteins USP18 and SOCS1, which synergistically act on the receptors and induce pathway desensitization. The IFNα regimes that lead to pathway hyper- or desensitization were shown to be patient-specific and controlled by the basal abundance of pathway components. Microfluidics time-lapse microscopy experiments in combination with mathematical modelling confirmed these results in non-liver cells (HeLa) [35]. In these studies, the duration of the stimulus rather than the dose was varied. A short pre-stimulation was shown to induce hypersensitization via IRF9, while a long pre-stimulation caused desensitization via USP18. Thus, hepatocytes possess a cellular memory of experienced environmental signals established by positive and negative feedback loops [36].

However, in the liver, hepatocytes not only encounter one ligand, but a multitude of different cues. For example, cells react to IFNα and IFNβ in a specific way, even though the exactly same receptors and intracellular signal transduction proteins are employed [37]. While both IFNα and IFNβ lead to the induction of antiviral genes, IFNβ is much more potent in inducing an anti-proliferative response. Interestingly, this phenomenon cannot be compensated by higher concentrations of IFNα. However, by mutating IFNα biochemically to increase its affinity to IFNAR1, this variant resembles IFNβ in its phenotype [38]. These results confirmed that the affinity of the interferons to the receptor subunit IFNAR1 is decisive for the induced cellular responses. However, it remained unclear how the JAK/STAT signal transduction pathway can decode these quantitative differences in affinity.

In a recent simulation study, this question was treated as an inference problem in information theory. The receiving cell should be able to discriminate between interferon IFNα, IFNβ or the absence of both ligands [39]. The simulations revealed that to optimally do this, the receptor system should comprise (i) heterodimeric receptors with (ii) asymmetric binding of the ligands to each receptor chain and (iii) receptor turnover. Interestingly, the organization of the interferon pathway is in line with all these proposed features. However, experiments have shown that the interferon pathway does not only qualitatively discriminate between the ligands, but also quantitatively responds differently to specific ligand doses [40]. By combining diverse data sets from different experimental systems using a minimal computational model, it was recently shown that while these differences in ligand binding strengths are theoretically sufficient for ligand discrimination, experiments failed to show a region of absolute discrimination between IFNα and IFNβ [41]. Rather, the mathematical model based on previous experimental data [42] showed that the negative feedback loop via USP18 contributes to ligand discrimination. Ligand binding assays showed that USP18 binds to IFNAR2, and thereby reduces the ability to recruit IFNAR1 to form a ternary complex [42]. Therefore, the presence of USP18 would reduce the dose–response curve of IFNα — the ligand with lower affinity to IFNAR1 — rather than the one elicited by IFNβ. This ligand-specific desensitization was previously not only shown in cell culture with hepatoma cells, but also *in vivo* by repeated interferon injections in mice followed by immunoblot analysis of liver tissue [43]. Thus, pre-stimulation of the interferon system is not only preparing the hepatocyte for the next ligand wave, but also seems to enable ligand discrimination.

Cross-talk with other cytokines complicates the input–output relationships even more. Moreover, it is very difficult to experimentally determine all possible scenarios, even if only two cytokines are considered. For a comprehensive analysis, cells would need to be stimulated with one factor, with both factors simultaneously, pre-stimulated with the first factor and stimulated with the second and vice versa. Furthermore, because pre-stimulation can result in both desensitization or hypersensitization, different doses of both ligands and/or time periods between first and second stimulation are required. As a readout, the activation of the signal transduction pathway as well as the physiological cellular response should be recorded. Here, mathematical modelling provides tools to *in silico* simulate all these different conditions to zoom in on interesting scenarios, which are addressable by experimental validation. Exemplary for such an approach, the cross-talk of IFNγ with IL6 was interrogated with a simulation study [44]. The simulations predicted that co-stimulation with IFNγ with IL6 increased STAT1 and STAT3 activation compared with stimulations with individual ligands. As an explanation for this increase, the mathematical model predicted that the cytoplasmic and nuclear phosphatases, which dephosphorylate phosphorylated STAT1 and phosphorylated STAT3, became limiting upon stimulation with both ligands. Furthermore, pre-stimulation with IFNγ reduced IL6-induced STAT3 phosphorylation, mediated by SOCS3, while IL6 did not desensitize the IFNγ-induced STAT1 pathway.

In a study employing mathematical modelling based on experimental data, factors that enhance the antiviral response to IFNα were identified. Co-stimulation with IL6 and IL8 had only a minor impact on gene expression. However, combining IFNα with IL1β resulted in a higher antiviral gene response in the human hepatoma cell line Huh7.5. Consistently, viral replication was shown to be enhanced in mice lacking the receptor for IL1β [45].

**Figure 2.**
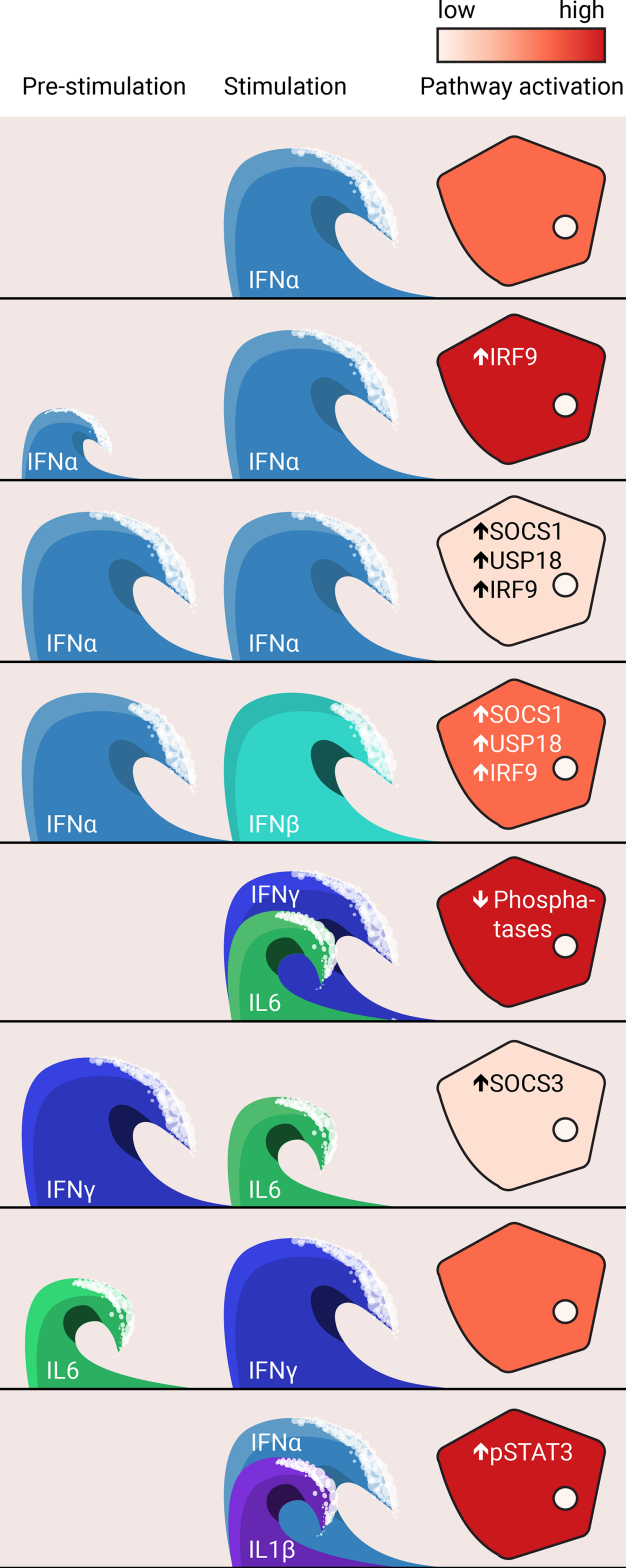
Cytokine waves augment or diminish pathway activation. Pre-stimulation and stimulation are indicated as consecutive waves, shades of red indicate the extent of activation of the JAK/STAT pathways. Data taken from references [34, 43–45].

In conclusion, as visualized in Figure 2, waves of cytokines augment or diminish pathway activation, depending on the order, dose and identity of the stimuli.

Temporal fluctuations are not restricted to cytokines in the liver. The secretion of growth factors and hormones, the metabolism and the cell cycle are all under periodic control, which may have implications for tumour growth and cancer therapy [46]. For example, the concentration of the peptide hormone insulin *in vivo* is not static, but displays rapid pulses of 10–15 min [47]. To investigate the decoding of these pulses by hepatocytes, rats were injected with different patterns of insulin while keeping glucose levels constant and blocking endogenous insulin secretion. These patterns included steps, ramp and pulses. The activation of the main components of the insulin-induced AKT pathway in the liver was measured by quantitative immunoblotting and qRT-PCR. An ordinary differential equation-based mathematical model was calibrated based on these data. Analysis of the mathematical model revealed that the information encoded by the temporal patterns of insulin was transferred to AKT without much loss of information. While this information was further transferred to GSK3β, S6K decoded information concerning the rate of change of insulin rather than its concentration. Contrary to this, the FoxO1 target gene G6Pase responded preferentially to basal secretion. Thus, combining quantitative data generation with mathematical modelling, it was shown that insulin patterns are selectively decoded by downstream molecules in the liver [48].

Major temporal fluctuations of hormone concentrations in the blood are encoded by circadian rhythms generated by the hypothalamo–pituitary–adrenal axis [49]. Endogenous glucocorticoid steroid hormones are secreted from the adrenal gland in a pulsatile manner with a circadian pattern. Therefore, plasma glucocorticoid concentrations are high during the active phase (day-time in humans and night-time in rodents), and low in the resting phase [50]. It was observed that synthetic glucocorticoids can inhibit EGF-mediated cell migration. Cell culture experiments revealed that glucocorticoid-induced signal transduction repressed EGF-induced gene expression. In line with this finding, it was demonstrated in mouse livers that positive and negative feedback regulators of EGF signal transduction display circadian oscillatory patterns *in vivo*. Thus, the authors concluded that the glucocorticoids enable circadian control of EGFR-induced signal transduction. During the active phase, EGF-induced cell fate responses are suppressed by high glucocorticoids, while they are enhanced during the resting phase [51].

In conclusion, cytokines and hormone concentrations in the liver are not static, but come and go in waves of varying amplitude and frequency. Depending on the order, dose and identity of the stimuli, cell fate decisions are augmented or diminished. Therefore, statistical analysis of static information is not sufficient, but dynamic mathematical analyses based on longitudinal data are required for decoding such multi-layered information.

## Interactions, trafficking and feedback regulations of receptors in the hepatocyte membrane

The liver is characterized by its remarkable capacity to regenerate upon injury such as partial hepatectomy and drug-induced liver injury [52]. Regeneration is triggered by several growth factors and cytokines, among which HGF plays a crucial role as it induces hepatocyte proliferation by activating its transmembrane receptor MET [53,54]. Numerous studies reported that MET deregulation by overexpression, activating mutations, splicing variants and amplification is implicated in tumour development and progression [55], positioning MET as therapeutic target for the treatment of cancer, including HCC [56]. Understanding the cell context-dependent mechanisms of HGF-induced activation of MET and its signal transduction pathways is essential to predict the cellular response to drug treatment. An example of such a cell context-dependent regulation is given by the interaction of MET with integrin α5β1. It was shown that reduction in integrin β1 by siRNA impaired hepatocytes proliferation and survival after partial hepatectomy [57] as well as HCC development [58]. The effect of integrin β1 on hepatocyte proliferation was associated with a lower activation of MET and EGFR in the former study and MET and β-catenin in the latter. A more recent study showed that the HGF-induced signal transduction was influenced by the treatment with the integrin inhibitor AXT050, an extracellular matrix derived mimetic peptide [59]. However, despite the knowledge of interactions between unrelated receptors at the cell surface, mechanistic understanding of the effect of such interactions is still missing. To investigate the impact of MET and integrin α5β1 interaction in response to drug therapy in HCC, Jafarnejad et al. [60] applied a systems biology approach. The authors implemented the interaction of MET with the integrin α5β1 in an ordinary differential equation-based mathematical model, which was established previously and described the HGF-induced cross-talk between the PI3K and MAPK signal transduction pathways [61]. The extended model considered that MET internalization, degradation and recycling is dependent on the interaction with the integrin α5β1, assuming that this interaction plays an important role in the regulation of HGF-induced signal transduction. This model was calibrated based on published data generated in primary mouse hepatocytes as well as in HCC cell lines, including the kinetics of protein phosphorylation and protein abundance [61,62]. Additionally, data generated in HCC cell lines treated with the integrin inhibitor, AXT050, and the Raf inhibitor, sorafenib, was used [59,63].

Based on the assumption that the interaction between MET and the integrin α5β1 influences MET internalization, degradation and recycling, the model predictions showed that the treatment with the inhibitor AXT050 strongly reduced MET activation as well as downstream signalling, such as AKT and ERK phosphorylation. Using the method of multidimensional synergy of combinations [64], the authors predicted the synergistic effect of co-treatment of AXT050 in combination with small molecule inhibitors against Raf or MET or with a monoclonal antibody directed against HGF. Such simulations that quantify the impact of a combined treatment with two inhibitors in comparison with the effect of a single compound facilitate prediction of the benefit of combinatorial therapy versus treatment with single drugs. Model predictions suggested that AXT050 has a synergistic effect on the early AKT activation when applied in combination with a potential HGF inhibitor and an additive effect on ERK phosphorylation. These observations were used to simulate the response to mono- and combination therapy in HCC patients based on TCGA data [65]. Since the model was calibrated on protein data, the authors extracted the mRNA information of the TCGA data and considered the fold-change of the mRNA as protein abundance of the HCC sample of each patient compared with the corresponding tumour-free sample. The model suggested that the interaction of MET with integrin α5β1 contributes to resistance mechanisms attributed to HGF signalling and that co-treatment of HCC cell lines with α5β1 inhibitor and RTK inhibitors inhibit the intracellular signalling response in a synergistic manner. This study is characterized by (i) accounting for signal transduction cross-regulation at the receptor and intracellular level; (ii) cell-type-specific experimental data; (iii) implementation of an already existing mathematical model of intracellular signal transduction cross-talk and (iv) translation of the mechanistic finding on patient-derived data.

Another prominent example of a growth factor that induces hepatocyte proliferation and is characterized by regulation at the receptor level is the EGF [66]. EGFR internalization can be triggered by clathrin-mediated endocytosis, involving the formation of vesicles coated by clathrin proteins, or by non-clathrin endocytosis. While at low doses of EGF clathrin-mediated endocytosis is the predominant mechanism and it is mainly involved in receptor recycling, at high EGF doses both internalization mechanisms are employed by the cell. The non-clathrin mechanism is linked to EGFR ubiquitination and therefore degradation of the receptor [67]. These observations indicate that clathrin-mediated endocytosis triggering EGFR recycling leads to sustained signalling, while non-clathrin endocytosis leads to termination by EGFR internalization and degradation, supporting the central role of receptor trafficking in signalling regulation. Mechanistically, the phosphorylation of specific tyrosine sites on EGFR is responsible for the recruitment of CBL, which binds to EGFR in complex with GRB2, and is necessary for EGFR ubiquitination [68]. Whereas tyrosine phosphorylation on EGFR linearly increased with increasing doses of EGF, EGFR ubiquitination and non-clathrin-mediated endocytosis showed a threshold response between 1 and 10 ng/ml of EGF. Capuani et al. [69] established an ordinary differential equation-based model aiming to understand the effect of the threshold response of non-clathrin internalization and receptor degradation on downstream signalling regulation. The model described EGFR activation by multisite phosphorylation events and receptor ubiquitination. First, the authors established a multisite phosphorylation model based on published data [68], representing the early EGFR phosphorylation mechanism. The applied model suggested that EGFR and phosphatases are not the limiting factors for the receptor phosphorylation and that all phosphorylation sites on EGFR have similar phosphorylation kinetics in the early activation after stimulation. CBL ubiquitinates EGFR via direct binding to phosphorylated EGFR or via complex formation with GRB2. Thus, to understand the ubiquitination process, in addition to EGFR and CBL, the authors experimentally measured the total amount of GRB2 in Hela cells. These measurements revealed CBL to be the rate-limiting factor regulating ubiquitination. Based on these results, the authors expanded the multisite phosphorylation model by implementing a module describing the ubiquitination process. The expanded model was used to understand the contribution of GRB2, CBL and EGFR in respect of the EGFR ubiquitination threshold by simulating a cooperative versus a non-cooperative interaction mechanism. These mechanisms are represented by the binding of CBL via a singly phosphorylated EGFR, of by binding through GRB2 to a doubly phosphorylated EGFR. Simulations showed that the singly phosphorylated EGFR is converted into the doubly phosphorylated form with increasing doses of EGF only with the cooperative interaction mechanism, suggesting that cooperativity is necessary for the ubiquitination threshold. It was previously shown that protein abundance can regulate the biological response [70] and it is known that EGFR is overexpressed in cancer [71]. The authors, therefore, utilized the model to make predictions to unravel the impact of EGFR abundance at the cell surface on its phosphorylation and ubiquitination upon a wide range of EGF doses. While phosphorylation increased with escalating doses of EGF, ubiquitination increased with lower numbers of EGFR molecules and at high EGF concentrations. This uncoupled mechanism between phosphorylation and ubiquitination was confirmed experimentally with cell lines expressing different levels of EGFR that were exposed with increasing doses of EGF. Because cancer cells expressed EGFR at high levels, the authors analysed cancer cell lines compared with fibroblasts and could confirm that the ubiquitination process is reduced in presence of a high abundance of EGFR. By employing the mathematical model, the authors showed that phosphorylation and ubiquitination of EGFR are uncoupled in lung cancer cells harbouring EGFR mutations without overexpression [72], suggesting that the impaired EGFR ubiquitination is enhancing EGFR signalling and therefore the tumourigenic characteristics. By mathematical modelling of the regulation of EGFR trafficking at the cell membrane, this study suggests that the interplay between the range of ligand, abundance and phosphorylation of the receptor expressed at the cell surface and intracellular regulators of internalization determine EGF-regulated downstream signal transduction.

The regulation of signal transduction pathways can take place at different levels, from the cell membrane with the regulation of receptor trafficking, to intracellular signalling cross-talk and finally to transcriptional regulation including feedback control of the signal transduction pathway. A prominent example of feedback regulation examined by a modular modelling approach is given by TNFα-induced signal transduction. TNFα is part of the inflammatory response upon liver damage and it is characterized by a wide spectrum of biological responses, ranging from pro-survival to pro-apoptosis [73]. In the liver, TNFα is secreted by macrophages upon liver damage and contributes to the initiation phase of liver regeneration by activating pro-survival signalling [74]. The mode of action of the negative feedback regulator A20 on the upstream signal transduction pathway has not been fully described and it is, therefore, formalized in different ways in mathematical models describing TNFα signal transduction. A20 can inhibit the inhibitor of NFκB kinase (IKK) directly or by interfering with the interaction between IKK and TNF receptor. Aiming to understand how the mode of action of A20 impacts TNFα signalling, Mothes et al. [75] compared the different A20 implementation modules of three published mathematical models of TNFα signal transduction [76–78]. The authors established an ordinary differential equation-based model (core model) describing NFκB binding to IκBα followed by its activation upon TNFα stimulation and IκBα degradation. Activated NFκB induces the transcriptional activation of IκBα, representing a negative feedback loop of known mechanism, and A20, whose mode of action on inhibiting NFκB can be formalized in different manners. To compare the mechanism of action of A20, the authors generated three modules describing different A20 regulation mechanisms upstream of the NFκB activation. Specifically, the authors compared the mode of action of A20 as follows: Model 1: A20 inhibits TNFα-dependent and TNFα-independent activation of IKK. Model 2: A20 inhibits the reaction of inactive IKK towards its neutral state, the so-called IKK form that can be activated by TNFα stimulation. This mechanism of action of A20 is dependent on TNFα stimulation. Model 3: A20 actives the TNFα-dependent transition of active IKK to inactive IKK. In this case, both the reaction neutral IKK towards active IKK and active IKK to inactive IKK are TNFα-dependent. To compare the impact of the three formalizations of A20, the core model was trained on the same experimental dataset [78] resulting in comparable protein kinetics among the three models. The effect of A20 on signalling dynamics was explored by predicting NFκB kinetics in combination with different strengths of A20 feedback alone and in combination with the other negative feedback regulations mediated by IκBα. The effect of the two negative feedbacks and their strengths was analysed on key features of signalling kinetics: (i) amplitude, (ii) peak time and (iii) signal duration. This analysis showed the impact of the combination of negative feedback regulators on signal transduction depending on their mode of action and feedback strength. In this specific study, only model 2 showed that the peak time of NFκB activation is regulated by IκBα, while A20 influences the NFκB signal duration in all three models. The advantage of using mathematical modelling resides in the possibility to test hypotheses that would not be possible to test experimentally. The combination of different strengths of A20 and TNFα was tested to analyse their impact on signalling features as described above. While the peak time and the amplitude of NFκB were influenced by the strength of TNFα in all models, the impact of A20 could be observed only in models 1 and 3. The authors showed that high TNFα increased NFκB signal duration in model 1, while in models 2 and 3 the same stimulation would cause a decrease in NFκB signal duration, suggesting that the different formalization of the mode of action of A20 in the three models affects the simulated predictions. Experimental validations of conditions tested with the model indicated that, at least in HeLa cells, model 1 represents the biological mode of action of A20.

**Figure 3.**
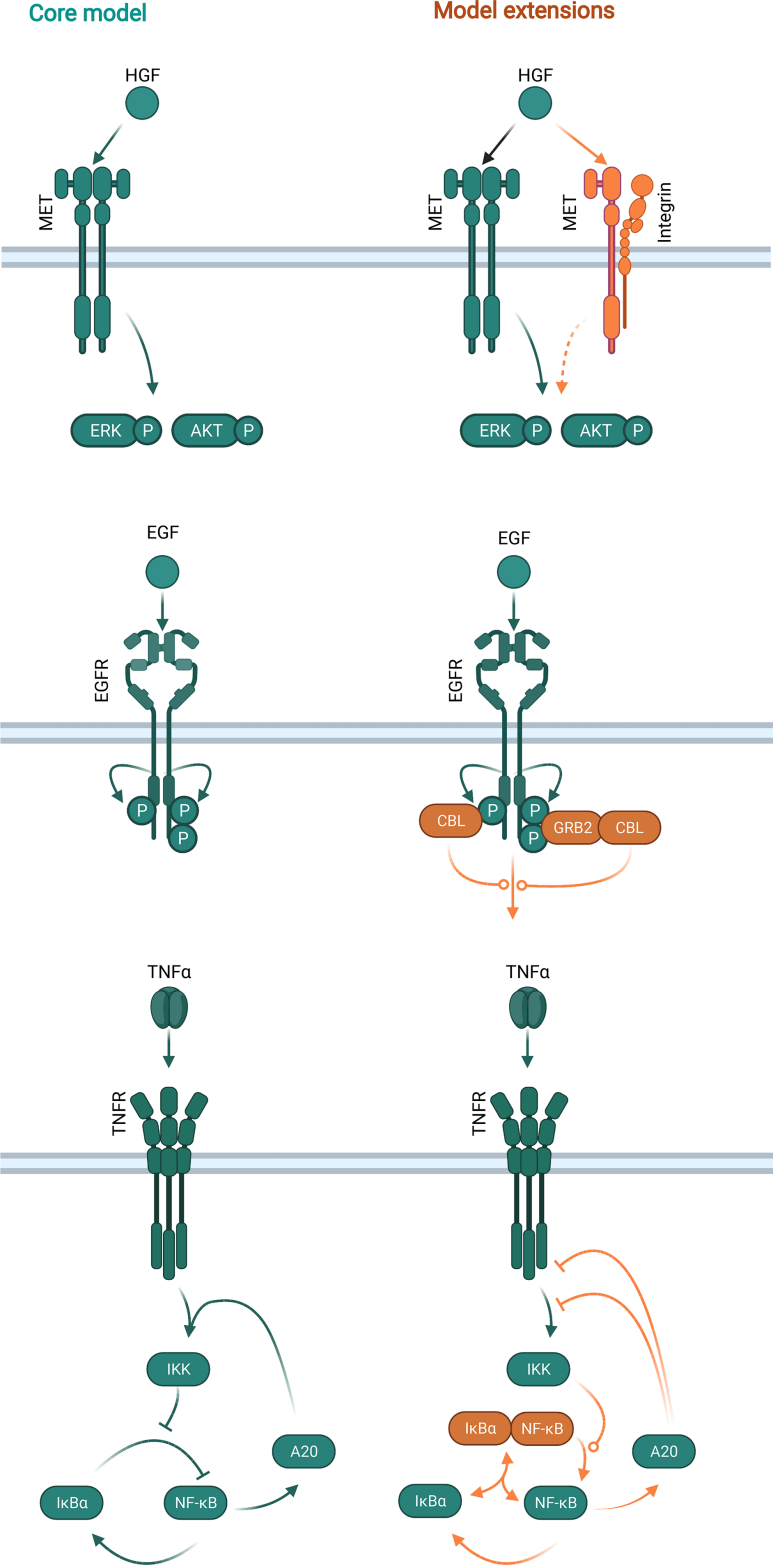
Modular approaches that were used for mechanistic mathematical modelling. On the left side, the core model is shown (dark cyan). On the right side, additional species and reactions that were used to extend the model are shown in orange. Top: HGF-induced ERK and AKT activation extended by interaction of MET with integrin α5β1 [60]. Middle: EGFR activation by multisite phosphorylation extended by CBL-based ubiquitination and internalization of EGFR [69]. Bottom: TNFα-induced NFκB signal transduction extended by specific A20 regulation mechanisms [75].

Taken together, these studies provide an overview of the complexity of the regulation of signal transduction at different levels. This complexity can be disentangled by mathematical modelling and quantitative experimental data. To identify the mode of interaction of signal transduction components, a modular modelling approach was shown to be of advantage (Figure 3). Additionally, it is important to note that in case of the HGF study presented here, the model has been established and validated in hepatocytes, therefore, can be regarded as a tissue-specific model. The EGF model suggests a general mechanism of receptor trafficking, allowing to adapt the model to different cell types. The TNFα model is an example of how modular modelling can be applied to identify a mode of signalling interaction, which can be cell type specific. Finally, it is noteworthy that mathematical models as well as experimental studies can be based on well-established models, which can be further developed to include new modules of a new level of signalling regulation or by integrating additional signal transduction pathways.

## Conclusions and future directions

In the last few decades efforts were undertaken to establish methods for the generation of mechanistic mathematical models and reliable parameter estimation based on quantitative experimental data [79]. These approaches allow understanding which parameters are mostly affected by perturbation [80] and enables the quantification of parameters that are difficult to measure experimentally [81]. Typically, the methodological development is performed on subsets of signal transduction pathways. In this review, we presented an improvement of dynamic modelling represented by modular approaches, allowing to accurately integrate different parts of a signal transduction pathway. However, methods for the integration of several signal transduction pathways still remain to be improved. The combination of different modelling approaches might be required to tackle the complexity of the signalling interaction among several pathways [61] and for the mechanistic modelling of single cell behaviour [82]. To be able to build mechanistic models of cell-to-cell communication, further methodological developments are necessary. For such a case, it was suggested to generate mechanistic models on the single cells, simplify these models by sampling their input–output behaviour and combine these as multi-cellular and multi-compartment models [83].

The examples for mechanistic modelling of signal transduction in liver cells discussed here frequently employed step-wise mathematical modelling using submodules. Signal transduction is a paradigm for a biological processes that is carried out by modules consisting of many species of interacting molecules [84]. It was quickly realized that it is of advantage to define modules during the establishment of a mathematical model. Especially, by defining the inputs and outputs of the modules, this approach allows to calibrate the individual modules separately based on experimental data [85]. The use of submodules is fostered by the re-use of both experimental data and mathematical models. Providing the experimental data to the community in public repositories such as the Gene Expression Omnibus [86] and the Proteomics Identification [87] database and as source data in publications [88] was shown to be extremely valuable. Similarly, progress in mathematical modelling was accelerated by public repositories for mathematical models, including JWS online [89] and the Biomodels database [90]. A precondition for such an exchange is the standardization of formats. For mechanistic mathematical models, the Systems Biology Markup Language (SBML) was widely adopted by the community [91]. Additionally, a unified format to support the mathematical model with data files describing the observation functions, experimental data and the parameters to be estimated was suggested, termed PEtab [92]. A recent study underscored the urgency of such standardizations by demonstrating that nearly half of the published mathematical models could not be directly reproduced given the information in the manuscript, mostly due to the lack of information [93]. To improve the reproducibility of mathematical models, a checklist was, therefore, suggested that would help to reproduce the simulation results. As mechanistic models grow in size, calibration of the mathematical model based on experimental data becomes more and more challenging. Therefore, efficient optimization tools for parameter estimation are required. Recently, a collection of benchmark problems ranging from 20 data points and 10 parameters to more than 1000 data points and 200 parameters to be estimated was presented [94]. This collection allows to evaluate model calibration algorithms by an unbiased assessment. Still, such data sets are small compared with approaches based on big data. However, because mechanistic models are theory-based, the model structure independent of the model parameters already confines the possible outputs. In contrast, the output of data-driven models used in the analyses based on big data are mostly determined by the data set used for machine learning [95]. In the future, we expect that hybrid approaches will show to be of advantage. For example, the simulated output of a mechanistic model describing signal transduction could be linked to phenotypic responses measured for various conditions by a machine learning approach.

The onset of liver cancer was reported to be frequently accompanied by changes in metabolic pathways, hinting to an intersection of metabolism and signal transduction in liver diseases [96]. Both, comprehensive metabolic reconstructions of the human hepatocyte [97] and detailed biochemistry-based kinetic models of liver metabolisms [98] have been developed. The regulation of metabolism regulation is interlinked with the immune response and intercellular interactions, requiring the integration of different aspects, such as genomic and proteome studies. While there are models linking genotype and metabolic phenotype [99], a unified framework for mechanistic mathematical models combining liver metabolism and signal transduction is still missing.

Apart from being applied to signal transduction and metabolism in the liver for basic research, mechanistic models disentangling intracellular complexity were employed for translational approaches. Mechanistic models were reported to predict the responses to drug treatment in NFκB signal transduction in hepatoma cell lines [100] and to simulate disease states of iron disorders in the liver [101]. Diabetes is a complex disease that is characterized by alterations in glucose metabolism. Treatment of diabetes with insulin is a prime example for mathematical modelling that can now be applied to suggest personalized dosing schemes. Several mathematical models have been established and applied to optimize insulin therapy [102]. The models describing glucose metabolism are continuously improved by inclusion of compartments representing different organs linked by the blood flow [103]. In the future, mathematical models promise to become part of clinical workflows to predict and optimize patient outcome after complex liver surgery [104]. In conclusion, mechanistic mathematical modelling was shown to be of advantage in deciphering liver signal transduction networks based on quantitative data. We expect that mechanistic modelling approaches will contribute to advance high-definition medicine [105], a data-driven practice of personalized medicine combining longitudinal and multi-parametric measurements to assess and manage health for the benefit of the individual patient.

## Abbreviations

EGF: epidermal growth factor
HBV: hepatitis B virus
HCC: hepatocellular carcinoma
HCV: hepatitis C virus
HGF: hepatocyte growth factor
IFN: interferon
IFNAR: interferon alpha/beta receptor
IKK: inhibitor of NFκB kinase
IL: interleukin
IL1RAP: IL1 receptor accessory protein
JAK: Janus kinase
MAPK: mitogen-activated protein kinase
NFκB: nuclear factor κ-light-chain-enhancer of activated B cells
PI3K: phosphoinositide 3-kinase
RTK: receptor tyrosine kinase
SBML: systems biology markup language
siRNA: small interfering RNA
SOCS: suppressor of cytokine signalling
STAT: signal transducer and activator of transcription
TNFα: tumour necrosis factor alpha
